# Polymorphisms in *PPAR* Genes (*PPARD*, *PPARG*, and *PPARGC1A*) and the Risk of Chronic Kidney Disease in Japanese: Cross-Sectional Data from the J-MICC Study

**DOI:** 10.1155/2013/980471

**Published:** 2013-11-07

**Authors:** Asahi Hishida, Kenji Wakai, Mariko Naito, Takashi Tamura, Sayo Kawai, Nobuyuki Hamajima, Isao Oze, Takeshi Imaizumi, Tanvir Chowdhury Turin, Sadao Suzuki, Motahare Kheradmand, Haruo Mikami, Keizo Ohnaka, Yoshiyuki Watanabe, Kokichi Arisawa, Michiaki Kubo, Hideo Tanaka

**Affiliations:** ^1^Department of Preventive Medicine, Nagoya University Graduate School of Medicine, 65 Tsurumai-cho, Showa-ku, Nagoya 466-8550, Japan; ^2^Department of Healthcare Administration, Nagoya University Graduate School of Medicine, Nagoya 466-8550, Japan; ^3^Division of Epidemiology and Prevention, Aichi Cancer Center Research Institute, Nagoya 464-8681, Japan; ^4^Department of Preventive Medicine, Faculty of Medicine, Saga University, Saga 849-8501, Japan; ^5^Department of Health Science, Shiga University of Medical Science, Otsu 520-2192, Japan; ^6^Department of Medicine, University of Calgary, Calgary, AB, Canada T2N 1N4; ^7^Department of Public Health, Nagoya City University Graduate School of Medical Sciences, Nagoya 467-8601, Japan; ^8^Department of International Island and Community Medicine, Kagoshima University Graduate School of Medical and Dental Sciences, Kagoshima 890-8544, Japan; ^9^Division of Epidemiology, Chiba Cancer Center Research Institute, Chiba 260-8717, Japan; ^10^Department of Geriatric Medicine, Graduate School of Medical Sciences, Kyushu University, Fukuoka 812-8582, Japan; ^11^Department of Social Medicine and Cultural Sciences, Kyoto Prefectural University of Medicine, Kyoto 602-8566, Japan; ^12^Department of Preventive Medicine, Institute of Health Biosciences, The University of Tokushima Graduate School, Tokushima 770-8503, Japan; ^13^Center for Genomic Medicine, RIKEN, Yokohama 230-0045, Japan

## Abstract

Chronic kidney disease (CKD) is well known as a strong risk factor for both end stage renal disease and cardiovascular disease. To clarify the association of polymorphisms in the *PPAR* genes (*PPARD*, *PPARG*, and *PPARGC1A*) with the risk of CKD in Japanese, we examined this association among the Japanese subjects using the cross-sectional data of J-MICC (Japan Multi-Institutional Collaborative Cohort) Study. The subjects for this analysis were 3,285 men and women, aged 35–69 years, selected from J-MICC Study participants; genotyping was conducted by multiplex polymerase chain reaction-based Invader assay. The prevalence of CKD was determined for CKD stages 3–5 (defined as eGFR < 60 ml/min/1.73 m^2^). Participants with CKD accounted for 17.3% of the study population. When those with *PPARD* T-842C *T/T* were defined as reference, those with *PPARD* T-842C *T/C* and *C/C* demonstrated the OR for CKD of 1.26 (95%CI 1.04–1.53) and 1.31 (95%CI 0.83–2.06), respectively. There were no significant associations between the polymorphisms in other *PPAR* genes and the risk of CKD. The present study found a significantly increased risk of CKD in those with the *C* allele of *PPARD* T-842C, which may suggest the possibility of personalized risk estimation of this life-limiting disease in the near future.

## 1. Introduction

Chronic kidney disease (CKD) is recently attracting attention as a leading cause of end-stage renal disease (ESRD) and a potential risk factor for cardiovascular disease (CVD). The prevalence of CKD is increasing over time in Japan, reaching about 22% of the adult population in 2002 [1–4]. Although the prevalence of CKD is shown to be specifically higher in Japan compared to those in other countries [5], it is also reported that about 10–15% of adults are affected by this disease in the developed Western countries [2], suggesting that CKD is becoming a general major public health problem worldwide, making its prevention a pressing universal issue.

Meanwhile, metabolic disorders are shown to play an important role in the genesis of CKD mainly through the mechanisms of insulin resistance, resultant hyperinsulinemia, and subsequent increase in plasma renin activity and plasma level of the renal vasoconstrictor angiotensin II [6]. While recent reports suggest that the MetS is an independent predictor of CKD development and progression [7], there are some other possible pathogenic factors causing CKD, such as smoking, hyperuricemia, and homocysteinemia [8]. Peroxisome proliferator-activated receptors (PPARs) are the firstly identified genetic sensor responsive to fatty acid ligands and ligand-activated transcription factors belonging to nuclear hormone receptor superfamily, named after its ability to bind chemicals known to induce peroxisome proliferation [9–11]. There are three PPAR families, PPAR-*α*, PPAR-*γ*, and PPAR-*δ*, each of which has distinct tissue distribution and they are also functionally at odds with each other [9]. These PPARs have been linked to many systemic and cellular functions ranging far beyond the process after which they were initially named; they function as the master regulators of glucose, fatty acid, and lipoprotein metabolism, energy balance, cell proliferation and differentiation, inflammation, and atherosclerosis [11, 12]. 

Compounds with agonistic activity on PPAR-*α*, such as fibrates, and PPAR-*γ*, such as thiazolidinediones, are widely used for the respective treatment of hyperlipidemia and insulin resistance. Although the activators of PPAR-*δ* are not in clinical use, emerging roles of PPAR-*δ* in regulating metabolism, inflammation, and antioxidant responses in various cell types and tissues argue in favor of the use of specific agonists to treat some aspects of metabolic dysfunctions [13]. Recently, PPARs have also been increasingly recognized as a key player in the pathogenesis of renal complications associated with metabolic disorders [14]. Meanwhile, there are a number of functional genetic variants in genes encoding PPARs, among which *PPARD* A65G (Asn163Asn: rs2076167) in exon 7, T-48444C (rs6902123) and T-842C (rs2267668) in exon 3, *PPARG* C161T (His477His: rs3856806) and Pro12Ala (rs1801282), and *PPARGC1A* Thr394Thr (rs2970847) and Gly482Ser (rs8192678) are the representative polymorphisms that are shown to modulate the function of PPAR pathways and studied well in association with the risks of various chronic diseases or health conditions [15, 16].

We conducted the Japan Multi-Institutional Collaborative Cohort (J-MICC) Study, a large genome cohort study to confirm and detect gene-environment interactions in lifestyle-related diseases, mainly cancer, launched in 2005 supported by a research grant for Scientific Research on Special Priority Areas of Cancer from the Japanese Ministry of Education, Culture, Sports, Science and Technology [17, 18].

Considering the potential roles of PPARs in various etiologies including metabolic disorders, inflammation, cell proliferation, and atherosclerosis, it would be plausible to hypothesize that genetic polymorphisms modulating the biological functions of PPARs will also affect CKD risk in humans. Accordingly, to clarify the association of polymorphisms in genes encoding PPARs (*PPARD, PPARG*, and *PPARGC1A*) with the risk of CKD in Japanese, we examined this association among the Japanese subjects using the cross-sectional data of this J-MICC Study.

## 2. Methods

### 2.1. Study Subjects

Subjects were the participants of the J-MICC Study, initially conducted in 10 areas of Japan, in which about 75,000 voluntarily enrolled participants aged 35–69 years provided their blood, health check-up data, and their lifestyle data based on the questionnaire after writting informed consent [17].

In this analysis, 4,519 randomly selected participants (about 500 subjects from each of the 10 areas) were analyzed, for whom 108 selected polymorphisms were genotyped [18]. Firstly, six subjects were excluded due to withdrawal from the study. Serum creatinine (SCr) was measured in 3,327 respondents from 8 areas out of 10. Twelve subjects were excluded because of genotyping failure, and the remaining 3,315 subjects were included in the analyses.

### 2.2. Evaluation of Lifestyle Exposure

Lifestyle exposures were evaluated with a self-administered questionnaire checked by trained staffs. The questionnaire included items on smoking status, alcohol consumption, and medical history. Smoking status was classified as current, former, or never, and the level of exposure was evaluated in pack-years. Former smokers were defined as people who had quitted smoking for at least 1 year. Alcohol consumption of each type of beverage was determined by average number of drinks per day and then converted into the Japanese sake unit: “gou” (180 mL), which is equivalent to 23 g of ethanol. 

### 2.3. Estimated Glomerular Filtration Rate (eGFR) and Definitions of CKD

Serum creatinine (SCr) was measured in all participants using an enzymatic method. The eGFR of each participant was calculated based on SCr, age, and sex using the following Japanese eGFR equation proposed by the Japanese Society of Nephrology [19]: eGFR (mL/min/1.73 m^2^) = 194 × SCr (mg/dL)^−1.094^  × age^−0.287^ (×0.739 if female). The prevalence of CKD was determined for CKD stages 3–5 (defined as eGFR <60 mL/min/1.73 m^2^).

### 2.4. Genotyping of Polymorphisms

DNA was extracted from buffy coat with a BioRobot M48 Workstation (QIAGEN Group, Tokyo). The genotyping of *PPARD *A65G in exon 7 (Asn163Asn) (rs2076167), *PPARD* T-48444C in exon 3 (rs6902123), *PPARD* T-842C in exon 3 (rs2267668), *PPARG* C161T (His477His) (rs3856806), *PPARG* Pro12Ala (rs1801282), *PPARGC1A* Thr394Thr (rs2970847), and *PPARGC1A* Gly482Ser (rs8192678) polymorphisms was conducted by the RIKEN institute using multiplex polymerase chain reaction-based Invader assay (Third Wave Technologies, Madison, WI, USA) as described previously [20]. The genotype distributions of all the 108 polymorphisms examined in this cross-sectional study are shown in the recently published data [18].

### 2.5. Statistical Analysis

Logistic regression analysis was performed for estimating age- and sex-adjusted odds ratios (aORs) and 95% confidence intervals (CIs) for CKD by genotype. All the other potential confounders of BMI, systolic blood pressure, diastolic blood pressure, use of anti-hypertensive drugs, fasting plasma glucose, HbA1c, use of glucose-lowering drugs, total cholesterol, HDL cholesterol, triglyceride, use of lipid-lowering drugs, uric acid, past history of cardiovascular diseases, past history of cerebrovascular diseases, smoking status, and drinking status were tested for change in estimate (CIE) [21] to see if any of these covariates produces significant change in estimates. We decided not to include any of these variables because none of them fulfilled the criteria of CIE ≥ 0.1. As it is known that metabolic factors such as HbA1c and presence of DM will affect CKD risk, they are considered to be causal intermediate of (*PPARD*) SNP and CKD risk. Adjusting for even a partially causal intermediate phenotype will incorrectly remove a true association and could potentially bias the true association [22]; thus we decided not to adjust for these variables. Gene-environment interactions were assessed by the logistic model, which included a multiplicative interaction term as well as variables for each genotype, age, sex, and smoking and drinking habits. Age adjustments in the analyses were done with ages regarded as continuous variables. Trend analyses by genotypes were done with genotypes for each polymorphism coded as ordinal-categorical variables. Differences in the distribution of the values of each characteristic variable between those with CKD and those without were examined by Student's *t*-test or by the *χ* ^2^ test. Accordance with the Hardy-Weinberg's equilibrium, which indicates an absence of discrepancy between genotype and allele frequencies, was checked using the *χ* ^2^ test. Haplotype analysis using genotypes in two loci was conducted by the “haplologit” command of STATA adjusted for age and sex based on the EM algorithm [23]. The linkage disequilibrium (LD) between the polymorphisms in two loci (*D*′ and *r* ^2^) was estimated by the “pwld” command of STATA. All the calculations were done using the STATA version 10 (Stata Corp, College Station, TX, USA). 

## 3. Results

### 3.1. Characteristics of the Subjects and Allele Frequency of the PPARD, PPARG, and PPARGC1A Polymorphisms

The characteristics of the subjects are summarized by CKD status in Table 1. The mean age ± standard deviation was 56.7 ± 8.6 years, and the males were 48.7% of the whole number of subjects. Subjects with CKD accounted for 17.3% (575/3,315) of the entire study population. 

The genotype frequencies among the genotyped subjects included in the analyses were in accordance with Hardy-Weinberg's equilibrium for all of the *PPARD *A65G (*G* allele = 0.221 (minor allele frequency), *χ* ^2^ = 0.020, and *P* = 0.8863), *PPARD* T-48444C (*C* allele = 0.020, *χ* ^2^ = 0.430, *P* = 0.5122), *PPARD* T-842C (*C* allele = 0.201, *χ* ^2^ = 0.017, *P* = 0.897), *PPARG* C161T (*T* allele = 0.152, *χ* ^2^ = 3.440, *P* = 0.0636), *PPARG* Pro12Ala (=*C/G*; *G* allele = 0.031, *χ* ^2^ = 0.213, *P* = 0.6445), *PPARGC1A* Thr394Thr (=*C/T*; *T* allele = 0.218, *χ* ^2^ = 0.642, *P* = 0.4228), and *PPARGC1A* Gly482Ser (=*G/A*; *A* allele = 0.462, *χ* ^2^ = 0.350, *P* = 0.5543). The allele frequencies were similar to those among all the genotyped 4,519 subjects: 0.215 for *PPARD *65*G*, 0.020 for *PPARD *−48444*C*, 0.195 for *PPARD *−842*C*, 0.152 for *PPARG* 161*T*, 0.031 for *PPARG* 12*Ala *(*G*), 0.219 for *PPARGC1A* Thr394Thr *T*, and 0.460 for *PPARGC1A* 482*Ser* (*A*) [18]. Genotype call rate was more than 99.8% for each genotype among those with SCr data (*n* = 3, 327).

### 3.2. PPARD, PPARG, and PPARGC1A Polymorphisms and Risk of CKD

When those with *PPARD* T-842C *T*/*T* (rs2267668) were defined as reference, those with *PPARD* T-842C*T*/*C* and *C*/*C* demonstrated the OR for CKD of 1.26 (95%CI 1.04–1.53) and 1.31 (95%CI 0.83–2.06), respectively, with the significant trend for increased OR with the increasing number of *C* allele (*P* = 0.018). There were no significant associations between the polymorphisms in other polymorphisms in genes encoding PPARs, *PPARD *A65G in exon 7 (Asn163Asn) (rs2076167), *PPARD* T-48444C in exon 3 (rs6902123), *PPARG* C161T (His477His) (rs3856806), *PPARG* Pro12Ala (rs1801282), *PPARGC1A* Thr394Thr (rs2970847), and *PPARGC1A* Gly482Ser (rs8192678), with the risk of CKD. When those with at least one minor allele of each* PPAR *SNP were combined together and compared with the references (subjects with homozygous major allele) (dominant model), those with *PPARD* T-842C *T*/*C* + *C*/*C* demonstrated the significantly increased risk of CKD with the aORs of 1.27 (1.05–1.53), while no other SNP turned out to be significant in this analysis (Table 2). The eGFRs by *GCK *and *GCKR* genotypes were substantially in the same trend to this analysis, with the marginally significant decreasing effect of *PPARD* T-842C*T*/*C* + *C*/*C* on eGFR, suggesting the influence of *GCK *and *GCKR* polymorphisms on human renal functions (Table 3).

**Figure 1:**
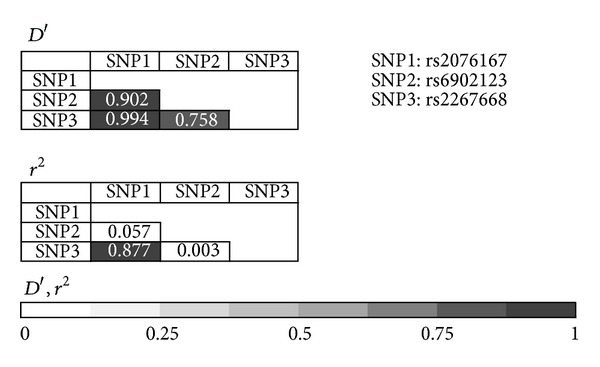
Linkage disequilibrium between the 3 *PPARD *polymorphisms.

Because one single nucleotide polymorphism (SNP) in *PPARD *gene (rs2267668) was significantly correlated with the CKD risk, we also estimated the LD within *PPARD* polymorphisms, which revealed that the 3 polymorphisms in *PPARD* gene investigated,* PPARD *A65G in exon 7 (Asn163Asn) (rs2076167), *PPARD* T-48444C in exon 3 (rs6902123), and *PPARD* T-842C in exon 3 (rs2267668), were in linkage disequilibrium to each other (Figure 1). Haplotype analysis of the *PPARD* polymorphisms on CKD risk did not reveal any significant association of *PPARD *haplotypes with the risk of CKD.

We also tested possible interaction between PPAR genotypes and lifestyle/etiologic factors including smoking, alcohol drinking, hypertension, dysglycemia, dyslipidemia, and high uric acid, none of which resulted in statistical significance (data not shown). As this study was held in 10 institutions, of which 8 had data for eGFR, we also conducted the analyses adjusted for institutions, the results of which were not substantially different from the unadjusted ones.

## 4. Discussion

To date, multiple factors have been identified to play key roles in the genesis of CKD induced by metabolic disorders, among which it is well recognized that insulin resistance and hyperinsulinemia are important factors [23]. In the pathogenesis of diabetic nephropathy patients, it is reported that renal structural features precede the appearance of overt diabetes, suggesting that renal injury is initiated in the phase of prediabetes [6]. In this process, the effect of insulin to stimulate both the renin-angiotensin system and nitric oxide in the renal vasculature is considered to be important [6]. PPARD plays an important role in energy homeostasis and overexpression of PPARD in skeletal muscle, and adipocytes are shown to increase fat catabolism in both of these tissues [24]. Given the important roles of FFA in the genesis of type 2 diabetes, the enhanced fatty acid oxidation as well as the upregulation of the oxidative phosphorylation pathway by PPARD may be beneficial in alleviation of insulin resistance and adiposity [24]. The fact that the PPARD agonists decrease insulin and glucose levels by increasing glucose transport suggests that genetic variations in *PPARD *gene will modify the glucose metabolism and possibly affect subsequent CKD risks. In the recent study, the rare single nucleotide polymorphisms (SNPs) of *PPARD* in LD with rs2267668 SNP and the *PPARGC1A* SNP were shown to have additive effects on the risk of type 2 DM, suggesting the etiological roles of this SNP also in the genesis of CKD [25]. The present study suggested that the polymorphism in the *PPARD* gene, a polymorphism in the gene encoding the key molecule controlling the expression of genes involved in fatty acid oxidation and energy uncoupling in skeletal muscles [26], is significantly associated with the risk of CKD in Japanese. As far as we know, this is the second report that investigated whether the genetic variations in *PPAR* genes may influence the renal functions, that is, the risk of CKD in Japanese [27], and the first one that indicated that the polymorphism in the *PPARD* gene may influence the risk of CKD in humans. It is also reported that common polymorphisms in *PPARD* gene were associated with fasting glucose, insulin resistance, and risk of conversion from impaired glucose tolerance to type 2 diabetes in Europeans [28], and some functional variants in *PPARD* gene were reportedly associated with fasting glucose and BMI in Koreans [29] and fasting glucose levels and insulin sensitivity in Chinese [30, 31]. Our study results suggested the trend that the subjects with the *C* allele of *PPARD* T-842C polymorphism were at an increased risk of CKD, which was in line with our hypothesis considering that the* PPARD* T-842C polymorphism found to be significantly associated with the risk of CKD in the present study is in tight linkage disequilibrium with other functional polymorphisms in *PPARD* gene, *PPARD* T294C (rs2016520) (*D*′ = 1.00 and *r* ^2^ = 0.81 in Japanese [JPT]) [32]. This functional polymorphism is located in the untranslated exon 4 of the *PPARD* gene influencing the expression of *PPARD *through altering the sequence of the DNA-binding site for Sp-1 [33, 34], and that demonstrated the possibility that the compromised function of PPARD due to this polymorphism might increase the risk of CKD as well as other vascular diseases. The *C* allele of the *PPARD* T-842C polymorphism is tightly linked to the *C* allele of the *PPARD* T294C polymorphism, carriers of which are shown to have a higher plasma LDL-cholesterol levels and it reportedly has a slightly higher promoter activity than the *T* allele in one *in vitro* study, which may promote lipid accumulation in macrophages as well as lipid uptake and storage [35]. The present study demonstrated that the subjects with the *C* allele of *PPARD* T-842C polymorphism were at an increased risk of CKD, which is considered to be biologically relevant, given the upregulation of lipid uptake/storage in the *C* allele carrier of the *PPARD* T294C polymorphism. However, considering that no evident associations between these *PPARD* SNPs and metabolic risk factors were observed in the present study, there seems to be a possibility that other factors such as inflammation are also involved in this CKD risk modification by the *PPARD* SNPs [36, 37].

Associations between polymorphisms in *PPARG* and risk of CKD including diabetic nephropathy (DN) are also reported; in a meta-analysis of 18 studies, it is demonstrated that the *Ala* allele carriers of the* PPARG* Pro12Ala polymorphism [38] had the reduced risk of DN, although this association was not significant when restricted only to Asians. Another study in Japanese showed that *PPARG* C-681G (rs10865710) polymorphism was significantly associated with the risk of CKD among subjects with hypertension, suggesting the biological roles of PPARs in the genesis of CKD also in Asian populations [27]. Regarding *PPARGC1A* polymorphisms, there is only one study that examined its association with DN in Asian Indians, which found that the Gly482Ser polymorphism of the *PPARGC1A *was significantly associated with DN [39]. Meanwhile, there are several studies that examined the association of these polymorphisms with the risk of type 2 diabetes (T2DM); the meta-analysis of the reported study results revealed that the *PPARGC1A* Thr394Thr polymorphism was not significantly associated with the T2DM risk in East Asian populations [40]. Our study results revealed no significant association of CKD risk with the polymorphisms in these genes encoding PPARs, *PPARG* C161T (His477His), *PPARG* Pro12Ala, *PPARGC1A* Thr394Thr, and *PPARGC1A* Gly482Ser, suggesting that the roles of these genetic variations are limited in Japanese, or the sample sizes of the present study were relatively underpowered to detect these associations.

A recent report from the STOP-NIDDM trial showed that the rare polymorphism of *PPARD* gene in linkage disequilibrium with the rs2267668 polymorphism was studied here and the 482Ser allele of the *PPARGC1A* rs8192678 polymorphism had the additive effects on the risk of type 2 diabetes [22], which makes the combined effects of these polymorphisms on the risk of CKD also an issue of interest. Although the present study results were considered to be in a similar trend for the effect of each of these polymorphisms on CKD risk, we could not detect any statistical interaction between these polymorphisms on the risk of CKD, which necessitates the investigation of this association with larger sample sizes in the near future. 

There are some potential limitations in this study. All of the CKD cases are diagnosed based on the SCr data, which might potentially be different from the actual GFR based on the renal measurement, and CKD diagnosis itself may also be different from that defined by the recent KDIGO 2012 Guideline, including the presence of albuminuria [41], which might have diluted the effect of each genotype on CKD risk. Different etiologies causing CKD should also have been considered in the analyses, but it could not be examined in the present study because of the limited availability of the clinical information including the pathologic one. Adjustments for multiple comparisons may be another issue. There are also a number of criticisms suggesting that correction of multiple comparisons by Bonferroni procedures is sometimes too conservative and aggravates the researchers' tendency not to conduct or present more tests [42], and considering that the present study was conducted under rather exploratory context, especially for the gene-environment interaction part, we decided not to adopt these adjustments in this study. Further investigations with better study designs will also be required.

In summary, the present study found a significantly increased risk of CKD in those with the *C* allele of *PPARD *T-842C, which may suggest the future possibility of personalized risk estimation of this life-limiting disease in the near future.

## Figures and Tables

**Table 1:** Comparison of characteristics between subjects with and without CKD (*N* = 3,315).

	CKD (+)	CKD (−)	*P* value
	(*n* = 575)	(*n* = 2,740)	
eGFR (mL/min/1.73 m^2^)	53.6 ± 6.1	78.3 ± 12.5	<0.001
Age (years)	60.5 ± 7.2	55.9 ± 8.7	<0.001
Male	268 (46.6%)	1,345 (49.1%)	0.280
Body mass index	23.5 ± 3.1	23.4 ± 3.3	0.507
Systolic blood pressure (mm Hg)	130.2 ± 19.8	128.1 ± 19.3	0.020
Diastolic blood pressure (mm Hg)	78.9 ± 12.4	78.6 ± 11.9	0.607
Use of antihypertensive drugs	155 (27.0%)	492 (18.0%)	<0.001
Fasting plasma glucose (mg/dL)	99.0 ± 22.1	100.0 ± 20.8	0.377
HbA1c (%)	5.22 ± 0.70	5.22 ± 0.66	0.885
Use of glucose-lowering drugs	33 (5.7%)	112 (4.1%)	0.078
Total cholesterol (mg/dL)	218.1 ± 33.8	211.0 ± 34.0	<0.001
HDL cholesterol (mg/dL)	62.0 ± 16.0	63.3 ± 16.3	0.086
Triglyceride (mg/dL)	106 (75–149)	104 (74–154)	0.832
Use of lipid-lowering drugs	72 (12.5%)	227 (8.3%)	0.001
Uric acid (mg/dL)	5.55 ± 1.49	5.11 ± 1.33	<0.001
Cardiovascular diseases	34 (5.9%)	79 (2.9%)	<0.001
Cerebrovascular diseases	31 (5.4%)	52 (1.9%)	<0.001
Current smokers	72 (12.5%)	492 (18.0%)	0.002
Current drinkers	302 (52.5%)	1,533 (55.9%)	0.133

Results are expressed as means ± standard deviation, *n* (%), or median (interquartile range). CKD: chronic kidney disease. CKD was defined as estimated glomerular filtration rate <60 mL/min/1.73 m^2^.

**Table 2:** *PPARD*, *PPARG,* and *PPARGC1A* polymorphisms and risk of CKD.

Polymorphism	Genotype	CKD (+)	CKD (−)	aOR (95% CI)	Trend P*
		(*n* = 575)	(*n* = 2,740)		
*PPARD* A65G in exon 7 (Asn163Asn) (rs2076167)	*A/A *	331 (57.6%)	1,681 (61.4%)	Reference	
	*G/A *	214 (37.2%)	929 (33.9%)	1.20 (0.98–1.45)	0.079
	*G/G *	30 (5.2%)	130 (4.7%)	1.20 (0.78–1.83)	
	*G/A* + *G/G *	244 (42.4%)	1059 (38.6%)	1.20 (0.99–1.44)	

*PPARD* T-48444C in exon 3 (rs6902123)	*T/T *	559 (97.2%)	2,628 (95.9%)	Reference	
	*C/T *	16 (2.8%)	110 (4.0%)	0.71 (0.41–1.22)	0.174
	*C/C *	0 (0.0%)	2 (0.1%)	0 (−)	
	*C/T* + *C/C *	16 (2.8%)	112 (4.1%)	0.70 (0.40–1.20)	

*PPARD* T-842C in exon 3 (rs2267668)	*T/T *	342 (59.5%)	1,776 (64.8%)	Reference	
	*C/T *	207 (36.0%)	855 (31.2%)	1.26 (1.04–1.53)	0.018
	*C/C *	26 (4.5%)	109 (4.0%)	1.31 (0.83–2.06)	
	*C/T* + *C/C *	233 (40.5%)	964 (35.2%)	1.27 (1.05–1.53)	

*PPARG* C161T (His477His) (rs3856806)	*C/C *	401 (69.7%)	1,968 (71.8%)	Reference	
	*C/T *	164 (28.5%)	719 (26.2%)	1.12 (0.91–1.38)	0.463
	*T/T *	10 (1.7%)	53 (1.9%)	0.88 (0.44–1.76)	
	*C/T* + *C/C *	174 (30.3%)	772 (28.1%)	1.10 (0.68–1.00)	

*PPARG* Pro12Ala (rs1801282)	*G/G *	535 (93.0%)	2,578 (94.1%)	Reference	
	*C/G *	38 (6.6%)	160 (5.8%)	1.17 (0.80–1.70)	0.216
	*C/C *	2 (0.3%)	2 (0.1%)	4.69 (0.62–35.51)	
	*C/G* + *C/C *	40 (6.9%)	162 (5.9%)	1.21 (0.84–1.75)	

*PPARGC1A* Thr394Thr (rs2970847)	*C/C *	372 (64.7%)	1,661 (60.6%)	Reference	
	*C/T *	170 (29.6%)	946 (34.5%)	0.79 (0.64–0.97)	0.223
	*T/T *	33 (5.7%)	133 (4.9%)	1.13 (0.75–1.70)	
	*C/T* + *T/T *	203 (35.3%)	1,079 (39.4%)	0.83 (0.68–1.00)	

*PPARGC1A* Gly482Ser (rs8192678)	*G/G *	162 (28.2%)	788 (28.8%)	Reference	
	*A/G *	275 (47.8%)	1,390 (50.7%)	0.96 (0.78–1.20)	0.151
	*A/A *	138 (24.0%)	562 (20.5%)	1.23 (0.95–1.59)	
	*A/G* + *A/A *	413 (71.8%)	1,952 (71.2%)	1.04 (0.85–1.27)	

aOR: adjusted odds ratio (adjusted for age and sex); CKD: chronic kidney disease.

*Adjusted for age and sex.

**Table 3:** *PPARD*, *PPARG,* and *PPARGC1A* polymorphisms and eGFR.

Polymorphism	Genotype	*n *	eGFR	*β*_dom⁡_*	*P* value	*β*_pa_**	*P* value
*PPARD* A65G in exon 7 (Asn163Asn) (rs2076167)	*A/A *	2,012	74.3 ± 14.8				
	*G/A *	1,143	73.4 ± 15.2	−0.607 ± 0.422	0.151	0.130 ± 1.153	0.911
	*G/G *	160	74.1 ± 15.0				

*PPARD* T-48444C in exon 3 (rs6902123)	*T/T *	3,187	73.9 ± 14.9				
	*C/T *	126	75.8 ± 15.9	1.458 ± 1.254	0.245	3.272 ± 10.070	0.745
	*C/C *	2	77.7 ± 17.4				

*PPARD* T-842C in exon 3 (rs2267668)	*T/T *	2,118	74.4 ± 14.7				
	*C/T *	1,062	73.2 ± 15.4	−0.826 ± 0.436	0.058	−0.320 ± 1.251	0.798
	*C/C *	135	73.9 ± 13.8				

*PPARG* C161T (His477His) (rs3856806)	*C/C *	2,369	74.2 ± 15.0				
	*C/T *	883	73.3 ± 14.4	−0.517 ± 0.495	0.296	1.727 ± 1.810	0.340
	*T/T *	63	75.4 ± 16.7				

*PPARG* Pro12Ala (rs1801282)	*C/C *	3,113	74.1 ± 14.9				
	*C/G *	198	73.1 ± 14.5	−1.303 ± 1.003	0.194	−8.380 ± 7.119	0.239
	*G/G *	4	64.5 ± 18.3				

*PPARGC1A* Thr394Thr (rs2970847)	*C/C *	2,033	74.0 ± 14.9				
	*C/T *	1,116	74.0 ± 14.7	−0.004 ± 0.420	0.993	−0.291 ± 1.134	0.797
	*T/T *	166	73.8 ± 16.7				

*PPARGC1A* Gly482Ser (rs8192678)	*G/G *	950	73.8 ± 15.1				
	*A/G *	1,665	74.0 ± 14.7	0.182 ± 0.352	0.606	0.302 ± 0.606	0.619
	*A/A *	700	74.3 ± 15.3				

eGFR: estimated glomerular filtration rate; data are shown in mean ± SD (standard deviation).

*β coefficient based on linear regression model (dominant model) adjusted for age and sex; data are shown in mean ± SE (standard error).

**β coefficient based on linear regression model (per allele model) adjusted for age and sex; data are shown in mean ± SE (standard error).
